# Glycogen synthase kinase-3β suppresses the expression of protein phosphatase methylesterase-1 through β-catenin

**DOI:** 10.18632/aging.102413

**Published:** 2019-11-12

**Authors:** Nana Jin, Ruirui Shi, Yanli Jiang, Dandan Chu, Cheng-Xin Gong, Khalid Iqbal, Fei Liu

**Affiliations:** 1 Key Laboratory of Neuroregeneration of Jiangsu and Ministry of Education of China, Co-Innovation Center of Neuroregeneration, Nantong University, Nantong, Jiangsu 226001, China; 2 Department of Neurochemistry, Inge Grundke-Iqbal Research Floor, New York State Institute for Basic Research in Developmental Disabilities, Staten Island, NY 10314, USA

**Keywords:** GSK-3β, PME-1, *LEF1/TCF cis*-element, β-catenin, methylation

## Abstract

Protein phosphatase 2A (PP2A) is the major tau phosphatase. Its activity toward tau is regulated by the methylation of PP2A catalytic subunit (PP2Ac) at Leu309. Protein phosphatase methylesterase-1 (PME-1) demethylates PP2Ac and suppresses its activity. We previously found that glycogen synthase kinase-3β (GSK-3β) suppresses PME-1 expression. However, the underlying molecular mechanism is unknown. In the present study, we analyzed the promoter of *PME-1* gene and found that human *PME-1* promoter contains two *lymphoid enhancer binding factor-1/T-cell factor* (*LEF1/TCF*) *cis*-elements in which β-catenin serves as a co-activator. β-catenin acted on these two *cis*-elements and promoted PME-1 expression. GSK-3β phosphorylated β-catenin and suppressed its function in promoting PME-1 expression. Inhibition and activation of GSK-3β by PI3K-AKT pathway promoted and suppressed, respectively, PME-1 expression in primary cultured neurons, SH-SY5Y cells and in the mouse brain. These findings suggest that GSK-3β phosphorylates β-catenin and suppresses its function on PME-1 expression, resulting in an increase of PP2Ac methylation.

## INTRODUCTION

Neuronal microtubule associated protein (MAP) tau is abnormally hyperphosphorylated and aggregated into neurofibrillary tangles (NFTs) in brains of individuals with Alzheimer’s disease (AD) and related neurodegenerative diseases [1, 2]. The number of NFTs in the brain positively correlates with the degree of dementia [3–5]. Hyperphosphorylation of tau not only destroys its biological activity but also converts it into a cytotoxic protein that sequesters normal tau and aggregates into NFTs [6–8]. Thus, hyperphosphorylation plays a pivotal role in tau pathogenesis in AD and related conditions.

Protein phosphatase 2A (PP2A) is the major tau phosphatase in human brain [9]. It dephosphorylates tau at multiple sites with various degrees of efficiency [9]. PP2A activity is reduced in AD brain [9, 10]. Inhibition of PP2A causes abnormal hyperphosphorylation of tau and AD-like tau pathology in cultured cells and *in vivo* in rodent brain [11–13]. PP2A consists of a core enzyme of scaffold A and a catalytic C subunit and a variable regulatory B subunit; the nature of the B subunit determines the localization and substrate specificity of the holoenzyme [14, 15]. Methylation of PP2A catalytic subunit (PP2Ac) at Leu309 is required for the association of core enzyme with PR55 B subunit to dephosphorylate tau [16–21].

Methylation level of PP2Ac is determined by protein phosphatase methylesterase-1 (PME-1) [22] and leucine carboxyl methyltransferase-1 (LCMT-1) [23]. We recently found that glycogen synthase kinase-3β (GSK-3β) suppresses PME-1 level, leading to increase of PP2Ac methylation [24]. However, the underlying molecular mechanism was unknown. In the present study, we found that β-catenin bound to the promoter of human PME-1 and enhanced PME-1 expression. GSK-3β phosphorylates β-catenin and suppresses its function, leading to decrease of PME-1 expression. These studies shed light on the molecular mechanism by which GSK-3β regulates PP2A methylation.

## RESULTS

### GSK-3β suppresses PME-1 expression

**Figure 1:**
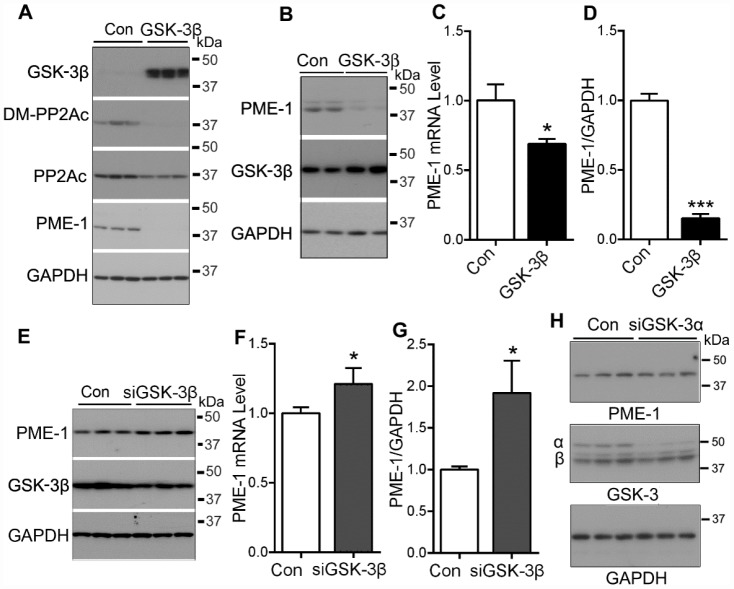
**GSK-3β suppresses the expression of PME-1.** (**A**) HEK-293T cells were transfected with pCI/HA-GSK-3β and the levels of GSK-3β, demethylated PP2Ac and PME-1 were analyzed by Western blots. (**B**–**D**) SH-SY5Y cells were transfected with pCI/HA-GSK-3β. The mRNA level of PME-1 was analyzed by qPCR (**C**). The protein levels of PME-1 and GSK-3β were analyzed by Western blots (B) and normalized with GAPDH (**D**). (**E**–**G**) SH-SY5Y cells were transfected with siGSK-3β to knockdown the expression of GSK-3β. The protein levels of PME-1 and GSK-3β were analyzed by Western blots (**E**) and normalized with GAPDH (**G**). The mRNA level of PME-1 was measured by qPCR (**F**). (**H**) SH-SY5Y cells were transfected with siGSK-3α to knockdown the expression of GSK-3α. The protein levels of PME-1 and GSK-3α/β were analyzed by Western blots. Data are presented as mean ± SD (n=3), *P < 0.05, ***P < 0.001.

We previously reported that PI3K-GSK-3β regulates PP2Ac methylation [24]. To confirm the role of GSK-3β in PP2Ac methylation, we overexpressed GSK-3β in HEK-293T cells and analyzed PP2Ac methylation by Western blots. Consistently, we found that the levels of demethylated PP2A and PME-1 were clearly reduced in GSK-3β overexpressed cells (Figure 1A), supporting that GSK-3β suppresses PME-1 expression and PP2Ac demethylation.

Then, we overexpressed GSK-3β in SH-SY5Y cells, and then measured the expression of PME-1 by Western blots and by quantitative real-time PCR (qPCR). We found that GSK-3β overexpression suppressed the expression of PME-1, as measured both at the mRNA level (Figure 1C) and the protein level (Figure 1B, 1D), suggesting that GSK-3β suppresses the expression of PME-1 in human neuroblastoma cells.

To further determine the role of GSK-3β on PME-1 expression, we knocked down GSK-3β by using siRNA and measured the PME-1 expression in SH-SY5Y cells. We found that knockdown of GSK-3β increased both the mRNA (Figure 1F) and protein (Figure 1E, 1G) levels of PME-1. However, knockdown of GSK-3α by its siRNA did not obviously affect the expression of PME-1 (Figure 1H). These results further confirm that GSK-3β inhibits PME-1 expression.

### Human PME-1 promoter contains two putative LEF1/TCF elements

**Figure 2:**
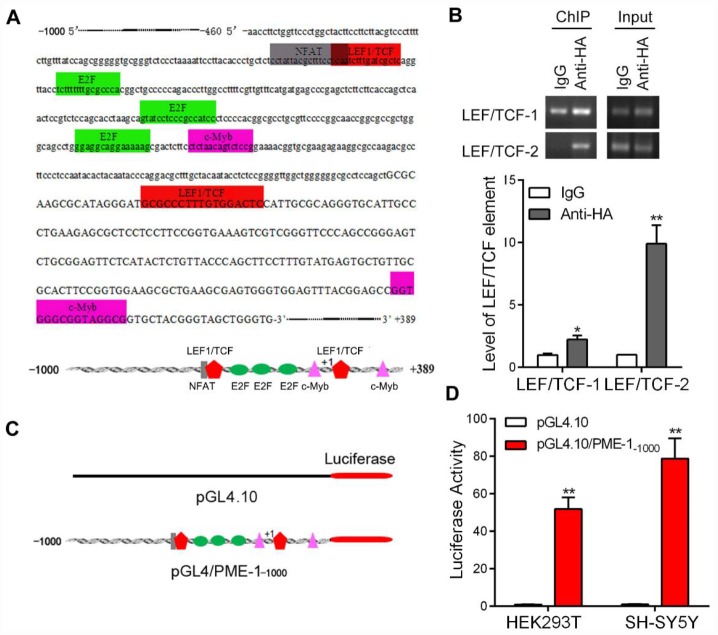
**The promoter of human PME-1 contains two putative *LEF1/TCF* elements.** (**A**) Human PME-1 promoter region has two potential *LEF1/TCF* like elements (red). The promoter (-1000 to +389) of human PME-1 was analyzed by MatInspector software. *Cis*-elements are labeled with different color. There are two potential *LEF1/TCF* like elements. Other *cis*-elements are NFAT (nuclear factor of activated T cells), E2F (transcription factor family including E2F- and DP-like subunits), c-myb (Cellular homologue of avian myeloblastosis virus oncogene). (**B**) The SH-SY5Y cells were overexpressed with β-catenin tagged with HA. Monoclonal anti-HA were used to immunoprecipitate β-catenin and co-immunoprecipitated LEF1/TCF cis-elements were amplified by PCR using primers specific to *LEF1/TCF cis*-elements 1 and 2. (**C**) Schematic diagram of PME-1 promoter and its luciferase reporter plasmid. Human PME-1 promoter was inserted into pGL4.10 containing luciferase reporter gene to generate pGL4/PME-1_-1000_. (**D**) pGL4/PME-1_-1000_ and pGL4.10 showed in panel **C** were transfected into HEK-293T or SH-SY5Y cells. The *Photinus pyralis* luciferase activity and *Renilla reniformis* luciferase activity were measured subsequently and the *Photinus pyralis* luciferase activity was normalized with *Renilla reniformis* luciferase activity. Data are presented as mean ± SD (n=3); *P < 0.05, **P < 0.01.

To understand how GSK-3β may suppress PME-1 expression, we first analyzed the promoter of the human *PME-1* gene by Genomatix’s MatInspector software [25, 26]. The bioinformatic analysis revealed an array of putative nuclear factor-binding sites, including two potential *LEF1/TCF* like elements located on -349 bp – -333 bp and +19 bp – +35 bp (Figure 2A), on which β-catenin acts as co-activator of TCF transcription factors to regulate the transcription of target genes.

To determine whether β-catenin binds to the *LEF1/TCF* elements of PME-1 promoter, we performed the ChIP assay in SH-SY5Y cells. We overexpressed β-catenin tagged with HA at the N-terminus in SH-SY5Y cells and immunoprecipitated β-catenin by anti-HA and then the bound DNA fragments were amplified by PCR to analyze co-immunoprecipitated *LEF1/TCF* elements of human PME-1 promoter. We found that anti-HA, but not control IgG, was able to co-immunoprecipitate two *LEF1/TCF* *cis*-elements 1 and 2 (Figure 2B), suggesting that β-catenin may regulate PME-1 expression through the *LEF1/TCF* elements.

To study the regulation of transcription of PME-1, we inserted the promoter region of human PME-1, -1000 to +389, into the pGL4.10 vector to generate the reporter plasmid, pGL4/PME-1^-1000^ (Figure 2C), transfected it together with pRL-TK into cells and measured luciferase activity by the dual luciferase assay. We found that the promoter of human PME-1 drove luciferase expression, leading to an increase of luciferase activity by ~50- and ~75 fold in the HEK-293T cells and SH-SY5Y cells, respectively (Figure 2D).

### *LEF1/TCF* *cis*-elements in PME-1 promoter enhance the PME-1 expression

**Figure 3:**
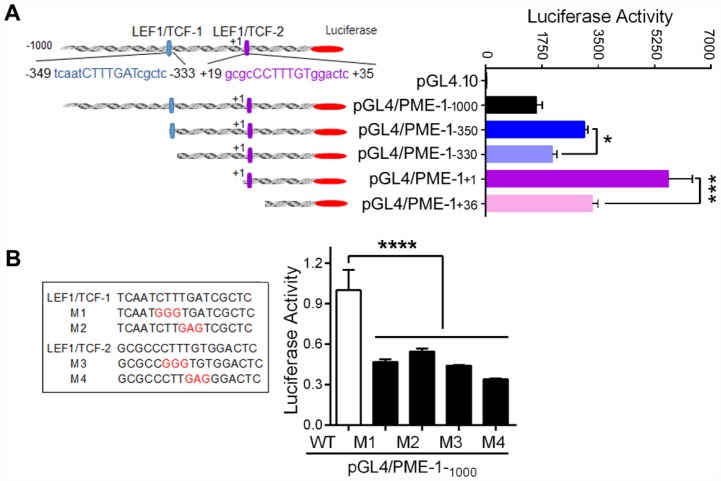
**LEF1/TCF cis-elements enhance the expression of PME-1.** (**A**) Schematic diagram of the sequential deletions of the human PME-1 promoter cloned into pGL4.10. Two LEF1/TCF elements are named as *LEF1/TCF*-1, located at -349 to -333 and labeled in blue color, and *LEF1/TCF*-2, located at +19 to +35 and labeled in purple color. The deletions shown as panel A left were co-transfected with pRL-TK into HEK-293T cells. The luciferase activity was measured. (**B**) *LEF1/TCF cis*-elements 1 and 2 were mutated and the mutated nucleotides are labelled in red color. pGL4/PME-1_-1000_ with different mutations were co-transfected with pRL-TK into SH-SY5Y cells. The luciferase activity was analyzed. Data are presented as mean ± SD (n=3), *P < 0.05, ***P < 0.001, ****P < 0.0001.

To investigate the role of two *LEF1/TCF* elements in PME-1 expression, we deleted *LEF1/TCF* elements of human PME-1 promoter in the pGL4/PME-1 (Figure 3A), and transfected them into HEK-293T cells, and analyzed luciferase activity to reflect the expression level of PME-1. We found that the luciferase activity was lower in pGL4/PME-1_-330_ than in pGL4/PME-1_-350_ and similarly it was lower in pGL4/PME-1_+36_ than that in pGL4/PME-1_+1_ (Figure 3A). Thus, deletion of both *cis*-elements 1 and 2 of *LEF1/TCF* dramatically suppressed the luciferase activity, suggesting *cis*-elements of *LEF1/TCF* work as enhancer and promote PME-1 expression. In addition, we found that deletion of -1000 bp – -350 bp or 330 bp – +1 bp enhanced luciferase activity, suggesting that these regions of PME-1 promoter may contain the suppressors, the nature of which remain to be determined.

To further verify the role of two *LEF1/TCF* elements in promoting PME-1 expression, we mutated two *LEF1/TCFs* (Figure 3B) and measured the luciferase activity. We found that M1, M2, M3 and M4 caused significant reduction of luciferase activity (Figure 3B), confirming that *LEF1/TCF* elements 1 and 2 both promote the expression of PME-1.

### β-catenin promotes the expression of PME-1 via *LEF1/TCF* *cis*-elements

**Figure 4:**
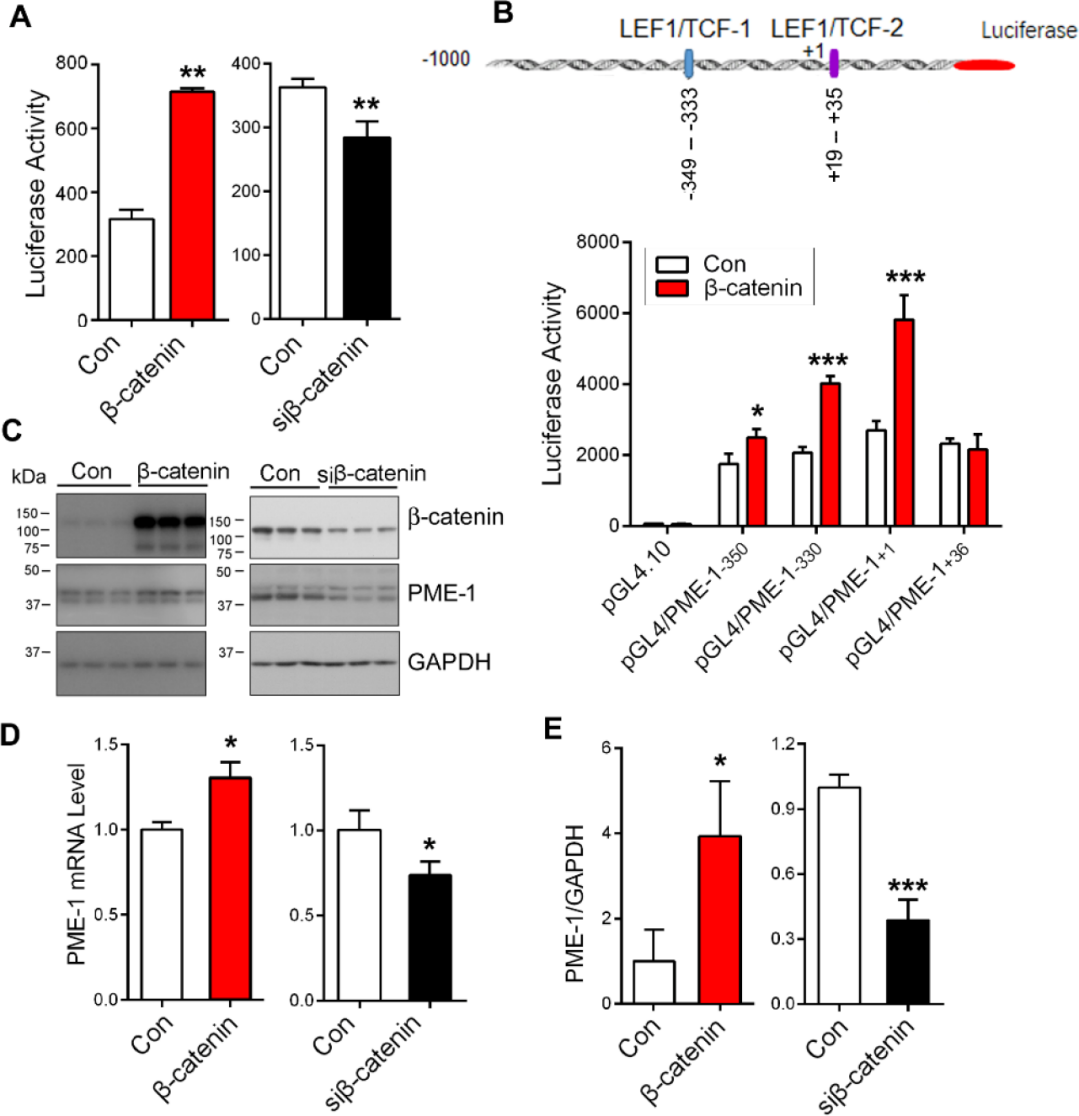
**β-catenin enhances PME-1 expression.** (**A**) β-Catenin or siβ-catenin was transfected together with pGL4/PME-1_-350_ and pRL-TK into HEK-293T cells, and then the PME-1 expression was determined through luciferase activity. (**B**) HEK-293T cells were co-transfected with pGL4/PME-1s, β-catenin and pRL-TK. The luciferase activity was measured. (**C**–**E**) HEK-293T cells were transfected with β-catenin or siβ-catenin and analyzed for PME-1 and β-catenin by Western blots (**C**, **D**) or qPCR (**E**). Quantification (**E**) of the Western blots are presented as mean ± SD. *P < 0.05, **P < 0.01, ***P < 0.001.

β-catenin acts as a co-activator of transcription factors TCF/LEF in regulation of target gene expression [27]. To evaluate the role of β-catenin in PME-1 expression, we co-transfected β-catenin or its siRNA (siβ-catenin) with pGL4/PME-1_-350_ in HEK-293T cells and measured the PME-1 expression using the luciferase reporting system. We found that β-catenin overexpression enhanced, and its knockdown suppressed the luciferase activity significantly (Figure 4A), indicating the regulation of PME-1 expression by β-catenin.

To verify the role of β-catenin in PME-1 expression by *LEF1/TCF* elements, we overexpressed β-catenin together with pGL4/PME-1_-350_, pGL4/PME-1_-330_, pGL4/PME-1_+1_, and pGL4/PME-1_+36_ and measured luciferase activity. We found that overexpression of β-catenin enhanced luciferase activity of pGL4/PME-1_-350_, pGL4/PME-1_-330_, and pGL4/PME-1_+1_, but not pGL4/PME-1_+36_ (Figure 4B). These results support that β-catenin acts on the two *LEF1/TCF* elements to promote the transcription of PME-1.

To determine the role of β-catenin in endogenous PME-1 expression, we overexpressed β-catenin or siβ-catenin in HEK-293T cells and analyzed PME-1 expression by qPCR and Western blots. We found that overexpression of β-catenin elevated the levels of endogenous PME-1 mRNA (Figure 4D) and protein (Figure 4C, 4E). Knockdown of β-catenin showed the opposite results in the protein (Figure 4C, 4E) and mRNA level (Figure 4D). Taking together, these results indicate that β-catenin also enhances the endogenous PME-1 expression.

### GSK-3β suppresses the function of β-catenin on PME-1 expression

**Figure 5:**
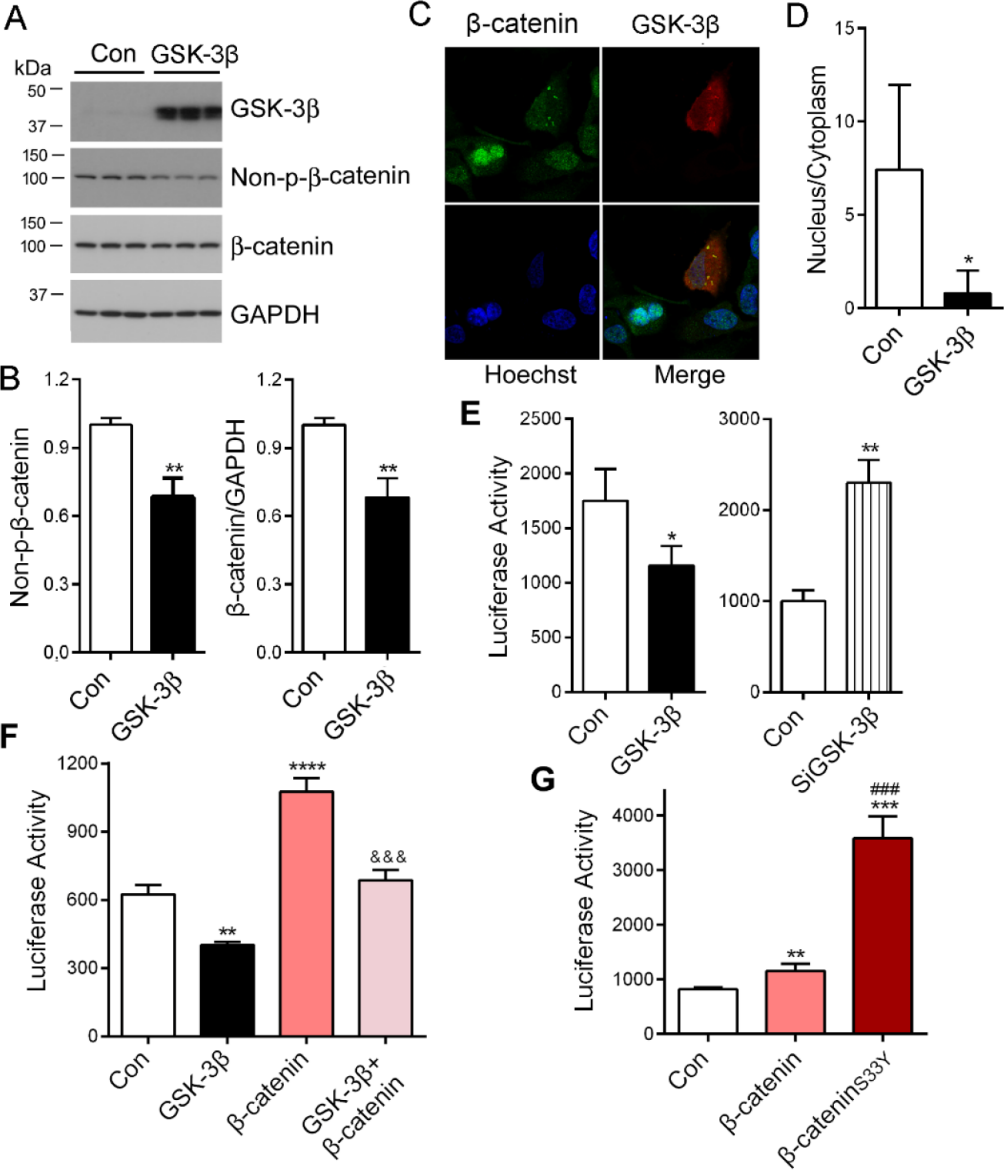
**PME-1 expression is regulated by GSK-3β/β-catenin pathway.** (**A**, **B**) HEK-293T cells were transiently transfected with GSK-3β. Levels of PME-1, β-catenin, GSK-3β, and Non-pS-β-catenin (dephosphorylated β-catenin at Ser33, Ser37 and Thr41) were analyzed by Western blots and normalized with GAPDH or corresponding proteins (**B**). (**C**, **D**) HA-tagged GSK-3β was overexpressed in HeLa cells and immuno-stained by polyclonal rabbit anti-β-catenin or mouse monoclonal anti-HA (GSK-3β) followed by florescence labeled anti-rabbit (green) or anti-mouse (red) second antibodies, respectively (**C**). The fluorescence levels of the nucleus and the cytoplasm were measured by IMAGE J and nucleus/cytoplasm ratio of β-catenin was analyzed (**D**). (**E**) pGL4/PME-1_-1000_ were co-transfected with GSK-3β or siGSK-3β in HEK-293T cells. The luciferase activity was measured. (**F**) HEK-293T cells were co-transfected with β-catenin and/or GSK-3β with pGL4/PME-1_-1000_. The luciferase activity was measured. *: compared with control (Con). &: compared with β-catenin. (**G**) The luciferase activity as measured. *: compared with control (Con), #: compared with β-catenin. Data are presented as mean ± SD (n=3), *P < 0.05, **P < 0.01, ***P < 0.001.

β-catenin is a known substrate of GSK-3β [28]. To investigate the role of GSK-3β in β-catenin-regulated PME-1 expression, we overexpressed GSK-3β in HEK-293T cells and analyzed β-catenin phosphorylation by Western blots and nuclear location by immunofluorescence staining. We found that overexpression of GSK-3β significantly decreased the levels of total and non-phosphorylated β-catenin at Ser33, Ser37 and Thr41 (Non-p-β-catenin) (Figure 5A, 5B) and suppressed its nuclear translocation (Figure 5C, 5D). Consistent with the previous study [29], these results suggest that GSK-3β phosphorylates β-catenin and suppresses its nuclear translocation.

To determine the effect of GSK-3β on PME-1 promoter activity, we co-transfected pGL4/PME-1_-1000_ with GSK-3β or its siRNA and analyzed the luciferase activity. We found that overexpression of GSK-3β significantly decreased the luciferase activity (Figure 5E), whereas knockdown of GSK-3β with siRNA increased the luciferase activity (Figure 5E). These results confirm that GSK-3β suppresses PME-1 transcription.

We co-expressed GSK-3β with β-catenin and determined the role of GSK-3β in β-catenin enhanced luciferase activity of pGL4/PME-1_-1000_. We found that luciferase activity was lower in the cells with co-overexpression of GSK-3β and β-catenin than that with β-catenin overexpression alone (Figure 5F). These results further suggest that GSK-3β suppresses β-catenin function in promoting PME-1 expression.

GSK-3β is known to phosphorylate β-catenin at Ser33 and promote its degradation by proteasome [28, 30, 31]. Missense mutation of Ser33 to Tyr in colorectal tumor cell line in SW48 is hyperactive as it avoids degradation and accumulates sufficiently to enter the nucleus [32, 33]. We mutated Ser33 to Tyr, β-catenin_S33Y_ and co-expressed it with pGL4/PME-1_-1000_. We found that compared with wild type β-catenin, β-catenin_S33Y_ increased the luciferase activity significantly (Figure 5G). Taking together, these results indicate that GSK-3β phosphorylates β-catenin and promotes its degradation, resulting in suppression of β-catenin function in the transcription of PME-1.

### Activation of PI3K/AKT signaling suppresses GSK-3β activity and promotes PME-1 expression

**Figure 6:**
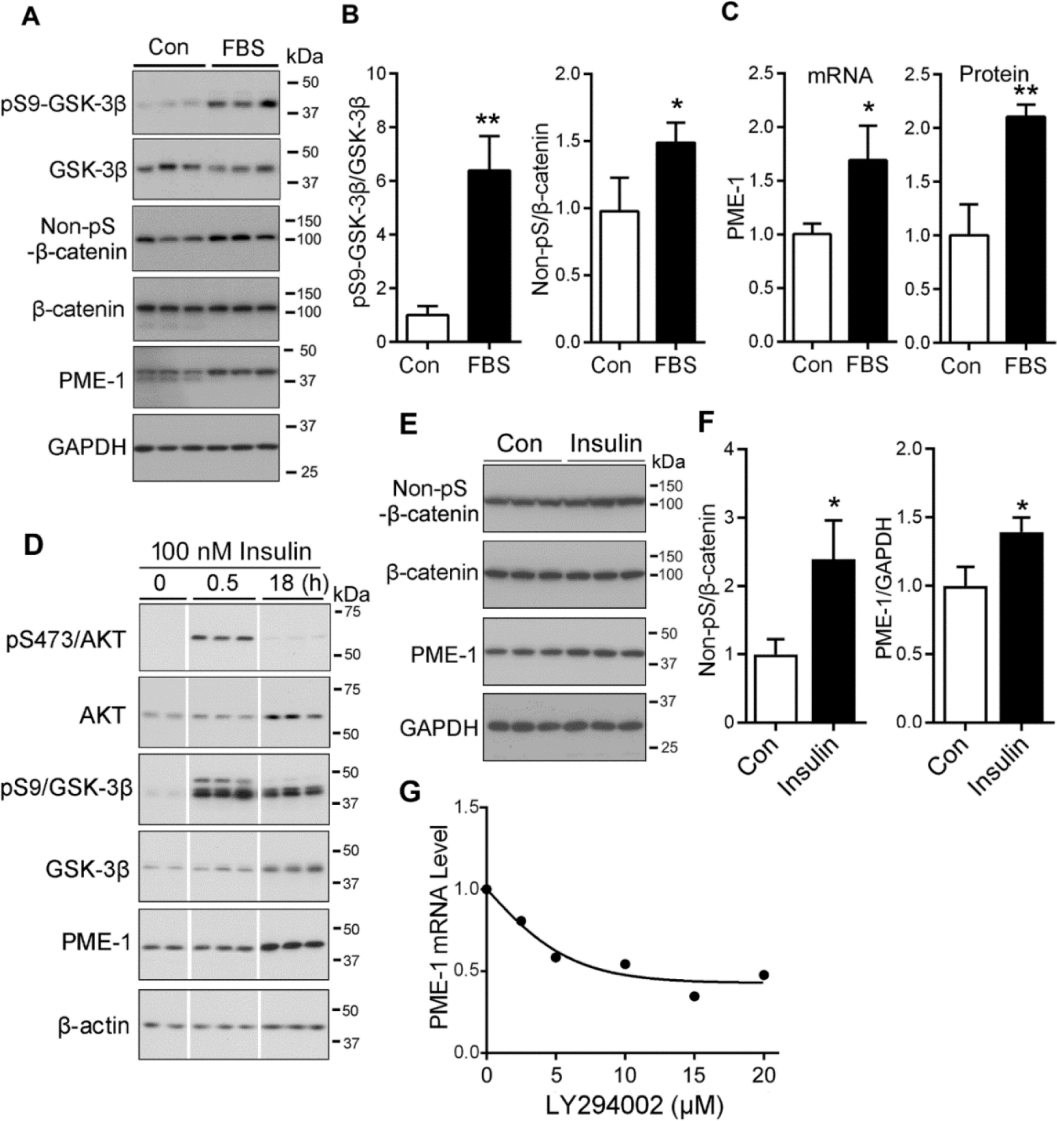
**PI3K/AKT signaling upregulates the PME-1 expression through GSK-3β/β-catenin.** (**A**–**C**) HEK-293T cells were transfected with β-catenin and cultured without Fetal bovine serum (FBS, as control, con) or with 2% FBS for 48 hr. The cell lysates were analyzed for total GSK-3β, β-catenin, PME-1 and the phosphorylation of β-catenin and GSK-3β by Western blots (**A**). Levels of phosphorylated β-catenin and GSK-3β were normalized with corresponding proteins (**B**). The mRNA level of PME-1 was measured by qPCR and normalized with GAPDH (**C**). The protein level of PME-1 was quantified from panel A and normalized with GAPDH. (**D**) SH-SY5Y cells were treated with 100 nM insulin for 0.5 hr or 18 hr and analyzed by Western blots developed with indicated antibodies. (**E**, **F**) Primary cortical neurons were treated with 100 nM insulin for 18 hr. The levels of β-catenin and PME-1 were analyzed by Western blots (**E**) and normalized with GAPDH (**F**). (**G**) Primary cortical neurons were cultured and treated with the indicated concentration LY294002 for 4.5 hr. The PME-1 mRNA level was measured by qPCR and normalized with GAPDH. Data are presented as mean ± SD (n=3); *P < 0.05; **P < 0.01.

Activation of PI3K-AKT signaling leads to inhibition of GSK-3β activity by phosphorylation at Ser9. To inhibit GSK-3β, we treated HEK-293T cells with 2% fetal bovine serum (FBS) for 24 hr to activate PI3K-AKT and analyzed the phosphorylation of GSK-3β and β-catenin by Western blots. We found that FBS induced the increase in phosphorylation of GSK-3β at Ser9 (Figure 6A, 6B) and increase of Non-pS-β-catenin (Figure 6A, 6B), compared with the cells cultured without serum. The levels of PME-1 mRNA and protein were increased in FBS treated cells (Figure 6C). Thus, inhibition of GSK-3β by activation of PI3K/AKT signaling suppressed β-catenin phosphorylation, leading to an increase in the expression of PME-1.

Insulin activates PI3K signaling and suppresses GSK-3β activity [34, 35]. To learn the regulation of PME-1 expression by PI3K-GSK-3β in neurons or neuronal like cells, following 2 hr culture without serum, SH-SY5Y cells were treated with or without 100 nM insulin for 0.5 hr or 18 hr and subjected to Western blots. We found that the phosphorylated Ser473-AKT and Ser9-GSK-3β were clearly increased in the cells treated with 100 nM insulin for 0.5 hr and /or 18 hr (Figure 6D), suggesting activation of PI3K signaling by insulin. The level of PME-1 did not obviously alter in the cells treated with 100 nM insulin for 0.5 hr, but was markedly increased in that for 18 hr (Figure 6D), supporting that inhibition of GSK-3β by insulin enhanced PME-1 expression in SH-SY5Y cells.

Then, we treated primary cultured neurons with 100 nM insulin for 18 hrs and analyzed PME-1 expression by Western blots (Figure 6E). We found that insulin treatment significantly increased the levels of non-pS-β-catenin and PME-1 (Figure 6F), confirming the enhancement of PME-1 expression by PI3K signaling in neuron.

Furthermore, we activated GSK-3β by inhibiting PI3K/AKT signaling with LY294002 in cultured neurons and analyzed PME-1 mRNA by qPCR. We found that LY294002 suppressed the expression of PME-1 in dose-dependent manner (Figure 6G), confirming that GSK-3β suppresses PME-1 expression in neurons.

### Specific inhibition of GSK-3β enhances PME-1 expression in cells and in vivo

**Figure 7:**
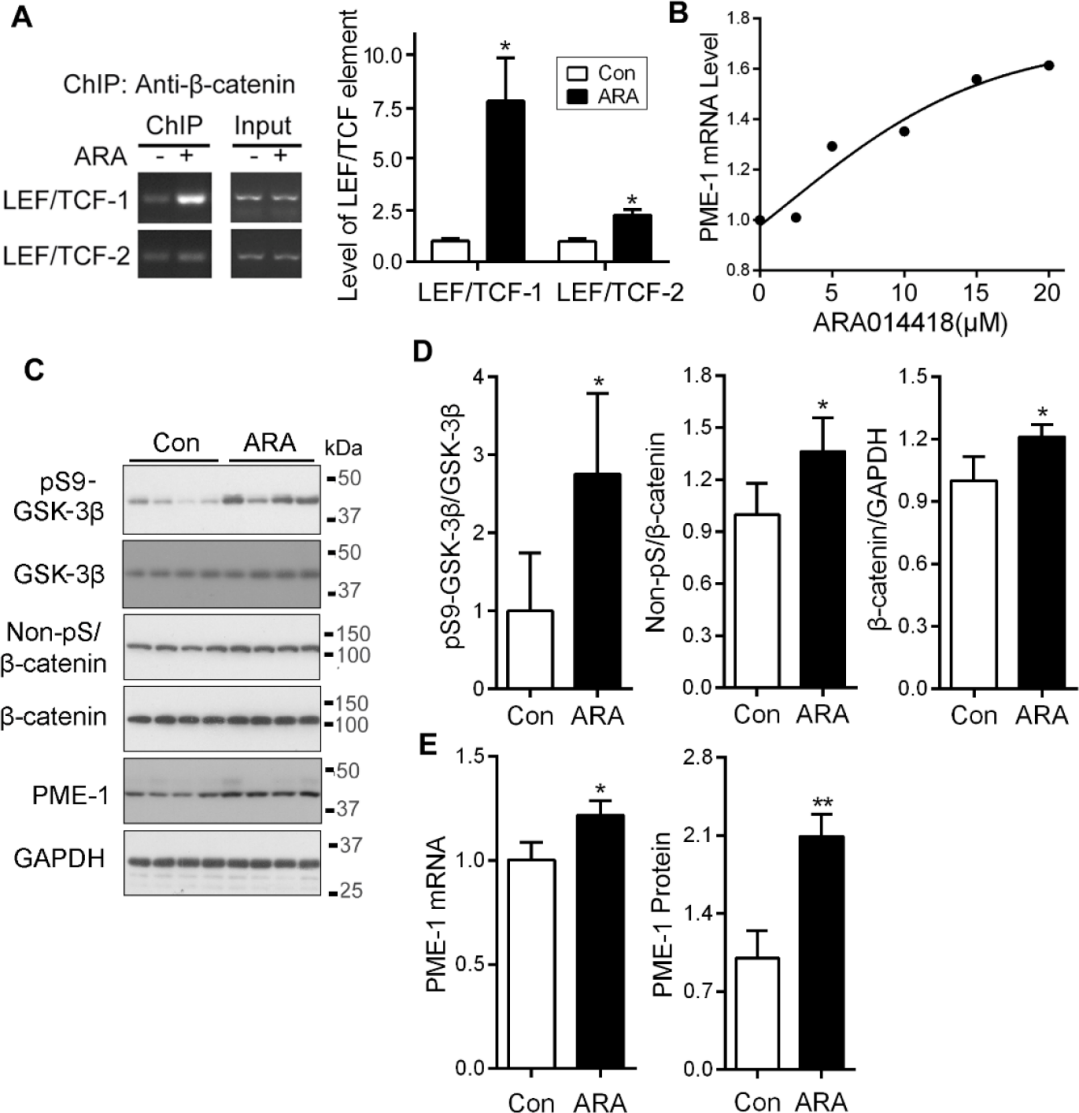
**Inhibition of GSK-3β with ARA014418 enhances the interaction of β-catenin with *LEF1/TCF* elements and up-regulates PME-1 expression in cultured cells and in vivo.** (**A**) SH-SY5Y cells were collected after ARA014418 (20μM) treatment for 4.5 hr, for ChIP assay using antibody to β-catenin. The two LEF1/TCFs were amplified by PCR with their specific primers. (**B**) Primary cortical neurons from embryonic day 18 SD rat were cultured and treated with the indicated concentration ARA014418 for 4.5 hr. The PME-1 mRNA level was measured by qPCR and normalized with GAPDH. (**C**–**E**) ARA014418 (5 mM 2 μl/mouse) was intracerebroventricularly injected into hTau transgenic mice for 48 hr. The cortex was homogenized and analyzed by Western blots developed with the indicated antibodies (**C**) or qPCR for PME mRNA (**E**). GAPDH was included as a loading control. Levels of phosphorylated β-catenin and GSK-3β were normalized with corresponding proteins (**D**). Data are presented as mean ± SD (n=4 or 5), *P < 0.05, **P < 0.01.

ARA014418 specifically inhibits GSK-3β activity by competing with ATP. We investigated whether inhibition of GSK-3β affects the binding of β-catenin with *LEF1/TCF cis*-elements. We overexpressed β-catenin in SH-SY5Y cells and treated the cells with the ARA014418 for 4.5 hr. We performed ChIP assay and found that ARA014418 treatment increased the levels of co-immunoprecipitated cis-elements of *LEF1/TCF*-1 and *LEF1/TCF*-2 by β-catenin (Figure 7A), suggesting inhibition of GSK-3β enhances the binding of β-catenin to *LEF1/TCF* elements.

To determine the effect of ARA014418 on PME-1 expression in primary cultured cortical neurons, we treated the neurons with various concentrations of ARA014418 for 4.5 hr and analyzed mRNA level of PME-1 by qPCR. We found that ARA014418 dose-dependently increased PME-1 mRNA level (Figure 7B).

To study the effect of GSK-3β on the expression of PME-1 *in vivo*, we injected 2 μl of 5 mM ARA014418 intracerebroventricularly in mice and analyzed the expression of PME-1 48 hr after injection by Western blots and qPCR in cerebral cortex. We found that ARA014418 increased levels of phosphorylation of GSK-3β at Ser9, non-phosphorylated β-catenin at Ser33, Ser37 and Thr41 and of total β-catenin (Figure 7C, 7D). Furthermore, the levels of PME-1 mRNA and protein were significantly increased in ARA014418 treated mice (Figure 7C, 7E). Thus, inhibition of GSK-3β enhances PME-1 expression *in vivo*.

## DISCUSSION

**Figure 8:**
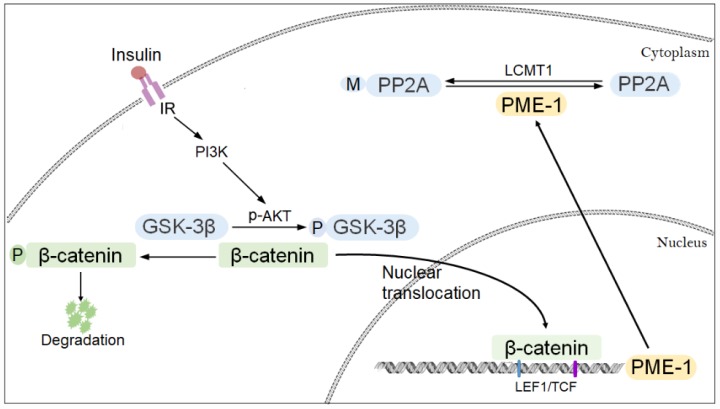
**Proposed model of regulation of PME-1 expression by PI3K-GSK-3β signaling.** Activation of PI3K signaling results in phosphorylation of GSK-3β and inhibition its activity in phosphorylating β-catenin. β-catenin translocates to nucleus and act as co-activator with LEF1/TCF to promote PME-1 expression, which catalyzes the demethylation of PP2Ac.

Methylation of PP2Ac plays an important role in the regulation of tau phosphorylation. We previously reported that GSK-3β enhances PP2Ac methylation through suppression of the expression of PME-1 [24]. In the present study, we further investigated the molecular mechanism involved in the regulation of PME-1 expression by GSK-3β. We found that the promoter of human PME-1 contains two *LEF1/TCF* cis-elements, which act as enhancers to promote the expression of PME-1 and that binding of β-catenin to *LEF1/TCF* *cis*-elements enhances the expression of PME-1. Furthermore, GSK-3β phosphorylates β-catenin and suppresses its nucleus translocation, leading to suppression of PME-1 transcription. Inhibition of GSK-3β by activated PI3K signaling through its phosphorylation at Ser9 enhanced PME-1 expression (Figure 8).

Transcription factors lymphoid enhancer-binding factors/T-cell factors (LEFs/TCFs) are known to bind to the consensus sequence CCTTTGWW, cis-element of LEF/TCF, and regulate the transcription of target genes [36]. In the present study, bioinformatic analysis with MatInspector revealed that the human PME-1 promoter contains two *LEF1/TCF* like elements: CTTTGAT at -344bp to -338bp and CCTTTGT at +23bp to +29bp. Deletion or mutation of each of *LEF1/TCF* motifs decreases significantly luciferase expression driven by human PME-1 promoter, suggesting that these two *LEF1/TCF cis*-elements promote PME-1 expression. In addition to *LEF1/TCF* *cis*-elements, PME-1 promotor contains several *cis*-elements, including NFAT, E2F, and c-Myb. Deletion of -1000 – -350 or -330 – +1 of PME-1 promoter significantly increased luciferase activity, suggesting *silencers* in these regions. The role of these *cis*-elements in the regulation of PME-1 transcription remains to be studied. The TCF/LEF is a group of transcription factors which bind to DNA through a high mobility group domain. The mammalian TCF/LEF family comprises four nuclear factors designated TCF7, LEF1, TCF7L1, and TCF7L2 (also known as TCF1, LEF1, TCF3, and TCF4, respectively) [37, 38]. These nuclear factors are involved in the Wnt signaling pathway, where they recruit the coactivator β-catenin to enhancer elements of the target genes [27]. Without Wnt stimulation, β-catenin forms a complex with axin (axis inhibitor), adenomatous polyposis coli (APC), casein kinase 1α (CK1α), and GSK-3β and undergoes phosphorylation-dependent ubiquitination and degradation [28, 30, 31, 39, 40]. Furthermore, conserved serine and threonine residues (Ser 33, Ser 37, Thr 41, and Ser 45) at the NH_2_-terminal domain of β- catenin are phosphorylated by GSK-3β and CK1α. Activation of Wnt signaling leads to the recruitment of Axin to the membrane and functional inactivation of the β-catenin destruction complex. This results in cytoplasmic and nuclear accumulation of β-catenin. In the nucleus, β-catenin associates with TCFs to activate transcription of Wnt signaling target genes. In the present study, ChIP assay showed that β-catenin bound to the two LEF/TCF cis-elements of PME-1 promoter and enhanced the PME-1 expression determined by using luciferase reporter.

Wnt/β-catenin signaling participates in diverse biological processes including neurogenesis [41], axonal remodeling [42, 43], formation and maintenance of pre- and post-synaptic terminals [44, 45], maintenance of blood brain barrier integrity [46], and excitatory synaptic transmission [47–50]. Perturbations of the Wnt/β-catenin signaling cascade were associated with Alzheimer’s disease (AD) onset and development [51, 52]. Reduced Wnt/β-catenin signaling was reported in AD brain and decreased expression of β-catenin was also found in AD patients carrying presenilin-1 (PS1) inherited mutations [41, 53]. In normal conditions, PS1 inhibits GSK-3β activity, enhancing β-catenin stability, through the stimulation of Akt. Conversely, PS1 mutations in AD patients are associated with increased GSK-3β levels [54–56] and low β-catenin levels which results in the inactivation of the Wnt/β-catenin pathway [57]. We previously reported that in AD brain, GSK-3β is upregulated as a result of its truncation by activated calpain [58]. GSK-3β is a major tau kinase that phosphorylates tau at multiple sites [59–61]. In the present study, we found that GSK-3β phosphorylates β-catenin and suppresses its function in promoting PME-1 expression, leading to an increase of PP2Ac methylation and hence PP2A activity. This increase in PP2A activity may attenuate the hyperphosphorylation induced by GSK-3β in brain in a site-specific manner.

PP2A is also known to regulate Wnt signaling by directly dephosphorylating β-catenin at Ser 37 and Thr41 to generate active β-catenin with enhances transcriptional activity [29]. PR55α, a PP2A regulatory subunit which requires methylated PP2Ac to form the holo- enzyme [62], regulates the dephosphorylation of β-catenin. Heat shock protein 105 (HSP105), a component of the β-catenin degradation complex, recruits PP2A to dephosphorylate β-catenin to maintaining a phosphorylation balance [63]. Thus, β-catenin may promote PME-1 expression, leading to demethylation of PP2Ac and hence decreased PP2A activity, which consequently may result in phosphorylation and reduction of β-catenin by a negative feedback.

In summary, we found here that β-catenin promotes PME-1 transcription. GSK-3β suppresses PME-1 expression by phosphorylation of β-catenin, leading to increase of methylation of PP2Ac. Thus, we speculate that activation of GSK-3β resulting from insulin resistant in AD brain one hand causes hyperphosphorylation of tau, other hand may increase PP2A methylation through PME-1, resulting in the attenuation of hyperphosphorylation of tau.

## MATERIALS AND METHODS

### Plasmids, proteins and antibodies

pCI/β-catenin and its mutants tagged with HA at N-terminus were constructed by PCR amplification from pcDNA3/β-catenin and confirmed by DNA sequence analysis. pGL4-1.0, pRL-TK (thymidine kinase promoter driven *Renilla reniformis* luciferase) and dual luciferase assay kit were bought from Promega (Madison, WI, USA). Luciferase driven by different length of or mutated promoter of human PME-1 in pGL4.10 were constructed by using human blood genomic DNA, purified by Spin Column Blood Genomic DNA Purification Kit (Sangon Biotech, China) as template and confirmed by sequencing. Monoclonal anti-PME-1, polyclonal anti-GAPDH, monoclonal anti-demethylated-PP2Ac (DM-PP2Ac), siGSK-3β, siGSK-3α and siβ-catenin were bought from Santa Cruz (Santa Cruz, CA, USA). Polyclonal anti-β-catenin, anti-Non-pS-β-catenin (dephosphorylated at S33/S37/T41), anti-GSK-3β and anti-pS9-GSK-3β were purchased from Cell Signaling Technology (Danvers, MA, USA). Monoclonal anti-HA was from Sigma (St. Louis, MO, USA). Horseradish peroxidase-conjugated anti-mouse and anti-rabbit IgG were obtained from Jackson ImmunoResearch Laboratories (West Grove, PA, USA). ECL (enhanced chemiluminescence) kit was from Thermo Fisher Scientific (Rockford, IL, USA).

### Animals

The hemizygous human tau transgenic (B6.Cg-Mapttm1(EGFP)Klt Tg(MAPT)8cPdav/J, Tg/hTau) mice with murine tau knockout background [64] obtained from the Jackson Laboratory (Bar Harbor, ME, USA) were used in this study. The mice were housed in cages at 24 ± 1 °C, 50~60% humidity under a 12 h light/dark cycle, with access to food and water ad libitum. The housing, breeding, and animal experiments were in accordance with the approved protocols from Institutional Animal Care and Use Committees of Nantong University and according to the United States PHS Policy on Humane Care and Use of Laboratory Animals.

### Cell culture and transfection

HEK-293T and human neuroblastoma SH-SY5Y cells were maintained in Dulbecco’s modified Eagle’s medium (DMEM) or DMEM/F12 supplemented with 10% fetal bovine serum (FBS) (Invitrogen, Carlsbad, CA, USA) at 37 °C, 5% CO_2_. All transfections were performed with Lipofectamine 3000 (Invitrogen, Carlsbad, CA, USA) according to the manufacturer’s instructions.

### Primary cortical neuron culture

Rat cerebral cortical neurons were isolated and cultured according to a previously described method [65]. Briefly, fetal cerebral cortices from Sprague Dawley (SD) rats at embryonic day 18 were cut into pieces in cold PBS buffer, digested with 0.25% trypsin at 37 °C for 15 min, and centrifuged at 1000 rpm for 5 min. The pellet was suspended in DMEM with 10% FBS. Following 400 mer filtering, cell density was counted and adjusted to 1 × 10^6^ cells/ml. The cells were plated onto poly-D-lysine (100 μg/ml) coated 12-well plates and were maintained in a humidified atmosphere at 37 °C, 5% CO_2_. The medium was changed to neurobasal supplemented with 2% B27 (Invitrogen) containing 100 U/ml penicillin and 100 μg/ml streptomycin 4 h later. On the second day of culture, the cells were treated with 10 μM Ara-C for 24 h to inhibit non-neuronal cells. On the seventh day, the cells were treated with various concentrations of ARA014418 or LY294002 at for 4.5 h for measurement of PME-1 mRNA or treated with 100 nM insulin for 18 hr for measurement of PME-1 protein.

### Quantitative real-time PCR (qPCR)

Total RNA was isolated from primary cultured cortical neurons or from cultured cells using the HP Total RNA Kit (Omega, Norcross, GA, USA). One microgram of total RNA was used for first-strand cDNA synthesis with Oligo-(dT)18 by using the HiScript II Q RT SuperMix for qPCR (Vazyme, Nanjing, JS, China). The target mRNA was amplified by using LightCycler® Multiplex DNA Masters (Roche, Indianapolis, IN) in a LightCycler® 96 system (Roche, Indianapolis, IN) under the condition: 95 °C 10 min, at 95 °C for 30 sec and at 60 °C for 1 min for 40 cycles. Relative levels of target mRNAs were calculated by the comparative CT (threshold cycle) method (2^-ΔΔCT^). The primers used for PME-1 are as follows: human PME-1 forward: 5′GTCGTCCTAAAACCTTCAAGTCTCT3′ and reverse: 5′CTCACTTCCTTCCTCATCTTCTTCT3′; mouse and rat PME-1 forward: 5′GAAGAAGATGAGGAAGGAAGTGAGT3′ and reverse: 5′AGAGCAGCAGTTTAGGAATAGGACA3′; human GAPDH forward: 5′GGTGGTCTCCTCTGACTTCAACA3′ and reverse: 5′GTTGCTGTAGCCAAATTCGTTGT3′; mouse GAPDH forward: 5′AGGTCGGTGTGAACGGATTTG3′ and reverse: 5’TGTAGACCATGTAGTTGAGGTCA3′; rat GAPDH forward 5′TGCACCACCAACTGCTTAGC3′ and reverse: 5'GGCATGGACTGTGGTCATGA3′.

### Dual luciferase assay

HEK-293T or SH-SY5Y cells were co-transfected with pCI/β-catenin, pCI/GSK-3β, pGL4/PME-1s with pRL-TK, and then the cells were lysed in 0.1 ml of passive lysis buffer (Promega, Madison, WI). The luciferase activity was measured by the dual luciferase assay (Promega, Madison, WI) according to manufacturer’s manuals. The *Photinus pyralis* luciferase activity and *Renilla reniformis* luciferase activity were measured subsequently and the *Photinus pyralis* luciferase activity was normalized with *Renilla reniformis*luciferase activity.

### Chromatin immunoprecipitation (ChIP)

SH-SY5Y cells were transfected with pCI/β-catenin tagged with HA at the N terminus and treated with or without 20 μM ARAO14418 for 4.5 hr. Chromatin immunoprecipitation (ChIP) assay was performed using the Simple ChIP® Enzymatic Chromatin IP Kit (Cell Signaling, Danvers, MA, USA), following the instructions of the manufacturer. The cells were fixed with 1% formaldehyde in PBS for 10 min at room temperature, and then glycine was added to a final concentration of 125 mM and incubated for 5 min at room temperature. The cells were collected in cold PBS with Protease Inhibitor Cocktail, then lysed with corresponding buffer. The nuclei were then collected and the chromatin was enzymatically digested into fragments ranging from 150 to 900 bp. ChIP was performed using protein G magnetic beads and either 2 mg of β-catenin antibody (Cell Signaling), HA antibody (Sigma), or the nonspecific IgG antibody (#2729; Cell Signaling). Protein-DNA cross-links were reversed, and the DNA was purified. The specific primers (listed in Table 1) were used to amplify regions containing *LEF1/TCF-1* and *LEF1/TCF-2* cis-elements. *LEF1/ TCF-1* region primer forward: 5′GTTTATCCAGCGGGGGTGCGGGTCT3′ and reverse: 5′GAAACAACGAAAAGGCCAAGGGTCTG3′; *LEF1/TCF-2* region primer forward: 5′ACGCTTTGCTACAATACCTCTCCG3′ and reverse: 5′CTGGGAACCCGACGACTTTCACCGG3′.

**Table 1:** List of primers used for generating mutated promoter of PME-1 constructs, mutated β-catenin constructs and TCF/LEF constructs.

**Construct**	**Forward primer**	**Reverse primer**
pGL4/PME-1_-1000_	5′cggggtaccatcccctactcggtgagcttgtgtctcct3′	5′ccgctcgaggaggtgcatgctcttttcgagggccg3′
pGL4/PME-1_-350_	5′ccgctcgagtgctctcctattacgctttcctc3′	5′ccgctcgaggaggtgcatgctcttttcgagggccg3′
pGL4/PME-1_-330_	5′ccgctcgaggttacctctttttttgcgcccacggc3′	5′ccgctcgaggaggtgcatgctcttttcgagggccg3′
pGL4/PME-1_+1_	5′ccgctcgaggcgcaagcgcatagggatgc3′	5′ccgctcgaggaggtgcatgctcttttcgagggccg3′
pGL4/PME-1_+36_	5′ccgctcgagcattgcgcagggtgcattgccctgaag3′	5′ccgctcgaggaggtgcatgctcttttcgagggccg3′
pGL4/PME-1_M1_	5′acgctttcctcaatcggggatcgctcaggttacctctt3′	5′gtaacctgagcgatccccgattgaggaaagcgtaataggag3′
pGL4/PME-1_M2_	5′ctttcctcaatcttgagtcgctcaggttacctctt3′	5′aggtaacctgagcgactcaagattgaggaaagcgtaatagg3′
pGL4/PME-1_M3_	5′atagggatgcgcccggggtggactccattgcgcagggtgcat3′	5′cgcaatggagtccaccccgggcgcatccctatgcgcttgcgc3′
pGL4/PME-1_M4_	5′tagggatgcgcccttgtgggactccattgcgcagggtgcat3′	5′tgcgcaatggagtcccacaagggcgcatccctatgcgcttgc3′
pCI/β-catenin_S33Y_	5′agtcttacctggactatggaatccattctggtgccactacc3′	5′caccagaatggattccatagtccaggtaagactgttgctgcca3′

### Immunofluorescence staining

HeLa cells were plated on glass coverslips in 24-well plates and transfected with pCI/HA-GSK-3β; 48 h later, the cells were washed with PBS and fixed with 4% paraformaldehyde in PBS for 30 min at room temperature. After washing with PBS, the cells were blocked with 10% goat serum in PBS containing 0.2% Triton X-100 for 1 hr at 37 °C, and incubated with mouse anti-HA (1:400) and rabbit anti-β-catenin antibody (1:400) overnight at 4 °C. After incubation with secondary antibodies (Alexa 488-conjugated goat anti-rabbit IgG and Alexa 555-conjugated goat anti-mouse IgG, 1:1000, Invitrogen, Carlsbad, CA, USA) plus Hoechst 33342 at room temperature, the cells were washed with PBS, mounted with SlowFade® Gold Antifade Mountant (Invitrogen, Carlsbad, CA, USA), and visualized with a TCS-SP5 dual photon laser-scanning confocal microscope (Leica, Bensheim, Germany). We used the Image J software to quantify the fluorescence intensity of the nucleus and whole cell. The ratio of fluorescence level in the nucleus and the cytoplasm (total-nucleus) was calculated and presented as average of the cells from 4–5 fields at random.

### Stereotactic injection

Mice were deeply anesthetized with 1.25% Avertin (Sigma, St. Louis, MO, USA) and placed in a stereotaxic apparatus (Stoelting, Wood Dale, IL, USA). After craniotomy, ARA014418 (2 μl of 5 mM dissolved in DMSO) was unilaterally injected into lateral ventricle of 6-month-old Tg/hTau mice using a 10 μl Hamilton syringe custom made with a 30-gauge/0.5 inch/ hypodermic needle (Hamilton Syringe Co., Reno, NV, USA). The coordinates were as follows: 0.3 mm posterior, 1.0 mm lateral to bregma, and 2.5 mm ventral to the dura surface. ARA014418 was injected at a rate of 0.66 μl/min, and the needle was kept in position for 3 min additionally before slow withdrawal to prevent leakage of the liquid infused. DMSO was injected into Tg/hTau mice of the same age by using the same approach as a vehicle control. The mice were allowed to completely recover on a soft heating pad before they were returned to their home cages. 48 hr after injection, mice were sacrificed by cervical dislocation. The cortex of mouse was homogenized as described above for Western blots and mRNA assay.

### Statistical analysis

The GraphPad Prism 5.0 software package was used for Statistical Analysis. The data are presented as mean ± SD. For multiple-group analysis, data points were compared by one-way ANOVA with Bonferroni post-hoc test. For two-group comparison, data points were compared by the unpaired two tailed Student’s *t* test.
